# COVID-19 among young adults in Sweden: self-reported long-term symptoms and associated factors

**DOI:** 10.1177/14034948211025425

**Published:** 2021-06-19

**Authors:** Sandra Ekström, Niklas Andersson, Alexandra Lövquist, André Lauber, Antonios Georgelis, Inger Kull, Erik Melén, Anna Bergström

**Affiliations:** 1 Center for Occupational and Environmental Medicine, Region Stockholm, Stockholm, Sweden; 2 Institute of Environmental medicine, Karolinska Institutet, Stockholm, Sweden; 3 Department of Clinical Science and Education, Södersjukhuset, Karolinska Institutet, Stockholm, Sweden; 4 Sachs’ Children and Youth Hospital, Södersjukhuset, Stockholm, Sweden

**Keywords:** COVID-19, young adult, Sweden, smoking, smokeless tobacco

## Abstract

**Aims:**

The main aim of the study was to describe self-reported symptoms of COVID-19 and examine if long-term symptoms are associated with lifestyle factors or common chronic diseases among Swedish young adults. A secondary aim was to compare the prevalence of smoking and snuff use before and during the COVID-19 pandemic.

**Methods:**

The study population includes 1644 participants aged 23–26 years from the Swedish population-based birth cohort BAMSE. From August to November 2020, the participants answered a web questionnaire on COVID-19 symptoms, lifestyle and health. Information on tobacco use was compared against the previous study follow-up in 2016–2019.

**Results:**

The prevalence of suspected COVID-19 symptoms was 45.3% (*n*=742), and 80 of these (10.8%) reported long-term symptoms (⩾4 weeks). There was no significant difference in sociodemographic or lifestyle factors in relation to the duration of suspected COVID-19 symptoms. Rhinitis, migraine and lower self-rated health before the pandemic was more common among participants with long-term symptoms. In addition, there was a tendency for higher prevalences of asthma, chronic bronchitis and depression in this group. The prevalence of smoking decreased from 18.9% before the pandemic to 14.7% during the pandemic, while snuff use increased from 12.7% to 22.4% (*P*<0.001).

**Conclusions:**

Almost half of Swedish young adults have had symptoms of suspected COVID-19 from February up to August 2020. Among these, one out of 10 have had long-term symptoms for at least 4 weeks. Long-term symptoms of suspected COVID-19 were associated with several common chronic conditions. Smoking may have decreased during the pandemic, while snuff use may have increased.

## Introduction

The COVID-19 pandemic poses a huge threat to public health, with severe pulmonary disease, increased mortality and risk of long-term health consequences in affected individuals. In Sweden, the disease was first confirmed on 31 January 2020, with the first death reported on 11 March. During the spring of 2020, COVID-19 started to spread rapidly in Sweden and many other parts of the world. Statistics from the Public Health Agency of Sweden show that there are now more than 1,000,000 confirmed cases and 14,000 deaths from COVID-19 in Sweden (up to 20 May 2021) [1].

The most important risk factor for COVID-19 mortality is age [2]. However, severe COVID-19 also affects younger adults and up to November 2020, 14% of the hospitalised COVID-19 patients in Sweden have been under 40 years of age [3]. In addition, there is increasing concern about long-term symptoms of COVID-19 (often referred to as ‘long COVID’ or ‘post-acute COVID-19’), which can involve multiple organ systems and also presents in younger individuals, including children [4–6]. In clinical studies, a large proportion of patients discharged from hospitals (33–87%) have reported post-acute symptoms (between 4 weeks to 6 months after disease onset) [4, 7], while there are few studies on long-term symptoms of COVID-19 in the general population. In the COVID Symptom Study, self-reported data from users of a mobile app in Great Britain, USA and Sweden showed that 13% of the COVID-19 cases (mean age 43 years) had symptoms for more than 28 days [5]. Reported long-term symptoms vary and may include fatigue, headache, cough, dyspnoea, joint and muscle pain, cognitive disturbances, loss of taste or smell and low-grade fever, which may relapse and remit [4, 5, 8]. These symptoms may also persist after a relatively mild disease [8, 9]. It is yet unknown why some individuals develop long-term symptoms from COVID-19. In addition to age, multiple chronic medical conditions and inflammatory or immune factors seem to play a role [8, 9]. Moreover, female sex, obesity and asthma were associated with long COVID (>28 days) in the COVID Symptom Study [5]. However, there is still limited knowledge about the long-term symptoms of COVID-19 and its potential risk factors among young adults.

Beyond the physical consequences of COVID-19, the pandemic may also affect mental health with increased worry, anxiety and depression [10]. Social isolation during the pandemic has been associated with increased substance use; that is, increased alcohol and drug consumption [11]. In contrast, tobacco smoking may have decreased after being revealed as a potential risk factor for severe COVID-19 [11], although social isolation, risk of unemployment and anxiety could make smoking cessation more difficult [12]. In Sweden, the use of smokeless tobacco (snuff) is common, especially among young adults [13]. Smokeless tobacco is generally considered as a less harmful alternative to smoking and is sometimes used during smoking cessation [14]. In a group of young adults in Sweden it is therefore of relevance to investigate if the patterns of both smoking and snuff use have changed in relation to the pandemic.

The main aim of the current study was to describe self-reported symptoms of COVID-19, and examine if lifestyle factors and common chronic diseases are associated with long-term symptoms of COVID-19 in a well characterised population of young adults from a Swedish birth cohort. As smoking was early highlighted as a potential risk factor for severe COVID-19, a secondary aim was to compare the prevalence of smoking and snuff use during the pandemic to recently collected information on these exposures prior to the pandemic (in 2016–2019).

## Methods

### Study population and study design

The study population includes participants from the prospective birth cohort, BAMSE, previously described in detail [15]. The BAMSE cohort originally included 4089 newborn children, recruited between 1994 and 1996 in the northwestern and central parts of Stockholm, Sweden. These have been subsequently followed with repeated questionnaires and clinical examinations including lung function testing and blood sampling. The latest follow-up was finished in 2019 when the participants were 22–24 years old years (referred to as the 24-year follow-up). The response rate was 74.9% for the questionnaire and 55.5% for the clinical investigation [16].

Participants who completed the clinical investigation at the 24-year follow-up study (*N*=2270) were invited to the current follow-up study. Starting 11 August 2020, an invitation letter was sent by email with information about the study and a link to a web questionnaire. The questionnaire was open to answer for 3 months (until 10 November 2020) and covered symptoms and testing of COVID-19 as well as lifestyle factors, occupation and living conditions during the COVID-19 pandemic from February 2020 until the date of answering the questionnaire. Out of the 2270 invited participants, 1644 (72.4% of invited) answered the questionnaire and were included in the current study population. The study was approved by the Swedish Ethical Review Authority (approval number 2020-02922). Participants provided written informed consent when answering the questionnaire.

### Definition of exposures and outcomes

Symptoms of COVID-19 were defined as having answered ‘Yes’ to the question ‘Have you had symptoms of suspected COVID-19 during the current pandemic (since February 2020)?’. Participants who answered ‘Yes’ to this question received follow-up questions on the type of symptoms, which month the symptoms started, for how long they lasted (‘Less than one week’, ‘1 week’, ‘2 weeks’, ‘3 weeks’, ‘4 weeks’, ‘5–7 weeks’, ‘8 weeks or more’) and if they were bed-bound or hospitalised due to the disease. The specific symptoms that were asked about were fever, cough, sore throat, runny nose, blocked nose, headache, joint or muscle pain, abdominal symptoms (stomach ache, nausea, vomiting and/or diarrhoea), breathing difficulties, tiredness, fatigue and other symptoms. Long-term symptoms of COVID-19 were defined as symptoms for at least 4 weeks, whereas short-term symptoms were defined as symptoms for less than 4 weeks.

Exposures and covariates were obtained from the current questionnaire and from the 24-year follow-up study. Smoking and snuff use were obtained from questionnaires at both follow-ups through the questions: ‘Do you smoke?’ and ‘Do you use snuff?’, respectively. The answer alternatives were: ‘No’, ‘No, but I have smoked before’/‘No, but I have used snuff before’ (referred to as ex-smoker and ex-user, respectively), ‘Yes, sometimes’, ‘Yes, every day’. Information on chronic diseases (asthma, chronic bronchitis, rhinitis, migraine, depression, attention deficit hyperactivity disorder (ADHD) or attention deficit disorder (ADD)) and self-reported health were obtained from the 24-year questionnaire.

Overweight (body mass index ⩾25 kg/m^2^), lung function, blood pressure and sensitisation to inhalant allergens were assessed during the clinical examination at the 24-year follow-up study. The details and definitions of these variables are described in the online Supplemental material.

### Statistical analyses

Descriptive data are presented as number (*n*) and percentage (%) for categorical variables and as mean and standard deviation (SD) for continuous variables. Differences between groups (women compared to men, long-term compared to short-term and no symptoms) were tested by chi^2^ test for categorical variables and by *t*-test or analysis of variance (ANOVA) for continuous variables. In order to evaluate the potential risk of selection bias, comparisons of baseline sociodemographic and lifestyle factors (see online Supplemental material for variables and definitions) were also performed between the study population and the remaining participants in the BAMSE cohort, using the chi^2^ test. A Wilcoxon matched-pairs signed-rank test was used to compare the prevalence of smoking and snuff use before (at the 24-year follow-up) and during (at the current follow-up) the pandemic. All analyses were performed using the statistical software Stata (Stata Corp., College Station, TX, USA) version 16.0. *P* values less than 0.05 were considered statistically significant.

## Results

### Description of the study population

The study population (*n*=1644) consisted of 996 women (60.6%) and 648 men (39.4%) and 1327/1644 (80.7%) lived in Stockholm county. Most of the participants (83.6%) answered the questionnaire in August, at a mean age of 25.3 years (Table I). Half of the participants (51.5%) reported working as their main occupation, while 37.8% were students. The prevalence of current tobacco use was 3.7% for daily smoking, 11.0% for occasional smoking, 13.5% for daily snuff use and 8.9% for occasional snuff use. There was no significant sex difference in occupation and smoking, while snuff use was more common among men (Table I).

**Table I.** Description of the study population, by sex (*n*=1644).

	Women (*n*=996)		Men (*n*=648)		*P* value^a^
	mean	SD	mean	SD	
**Age (years) (*n*=1644)**	25.3	0.78	25.3	0.80	0.79
	*n*	%	*n*	%	*P* value^b^
**Living area (*n*=1644)**					
**Stockholm county**	791	79.4	536	82.7	
**Outside of Stockholm county**	205	20.6	112	17.3	0.10
**Month of answering the questionnaire (*n*=1644)**					
August 2020	865	86.9	509	78.6	
September 2020	78	7.8	85	13.1	
October 2020	47	4.7	48	7.4	
November 2020	6	0.6	6	0.9	<0.001
**Occupation (*n*=1641)**					
Working	514	51.7	331	51.2	
Studying	374	37.6	246	38.0	
Other	106	10.7	70	10.8	0.98
**Current smoking (*n*=1641)**					
No	694	69.8	482	74.5	
Ex-smoker	138	13.9	85	13.1	
Yes, sometimes	118	11.9	63	9.7	
Yes, everyday	44	4.4	17	2.6	0.09
**Current snuff use (*n*=1644)**					
No	789	79.2	420	64.8	
Ex-user	38	3.8	29	4.5	
Yes, sometimes	86	8.6	60	9.3	
Yes, everyday	83	8.3	139	21.5	<0.001

a *P* value obtained from *t*-test.

b *P* values obtained from chi^2^ test.

Compared to the remaining participants in the BAMSE cohort, the study population consisted of more women and participants from households with somewhat higher socioeconomic status (in terms of living area at birth, type of parental work, lower maternal age and higher prevalence of maternal smoking), as well as fewer participants with parental origin outside of Sweden (Supplemental Table I).

### Description of self-reported symptoms of suspected COVID-19

The prevalence of symptoms of suspected COVID-19 was 45.3%, similar in women (46.2%) and men (43.8%), *P*=0.33. The five most common symptoms were tiredness, runny nose, headache, sore throat and fatigue, and women reported a higher number of symptoms than men (63.7% vs. 53.6% had seven or more symptoms) (Table II).

**Table II.** Description of type of symptoms, duration, time period and severity among participants with self-reported symptoms of suspected COVID-19 (*n*=742).

	Women (*n*=460)	Men (*n*=282)	Total (*n*=742)	*P* value^a^
	*n* (%)	*n* (%)	*n* (%)	
**Type of symptoms**				
Tiredness (*n*=736)	412 (90.1)	232 (83.2)	644 (87.5)	0.005
Runny nose (*n*=731)	323 (71.5)	209 (74.9)	532 (72.8)	0.31
Headache (*n*=730)	346 (76.2)	184 (66.7)	530 (72.6)	0.005
Sore throat (*n*=734)	339 (74.0)	184 (66.7)	523 (71.3)	0.03
Fatigue (*n*=734)	332 (72.8)	172 (61.9)	504 (68.7)	0.002
Cough (*n*=737)	285 (62.4)	203 (72.5)	488 (66.2)	0.005
Fever (*n*=712)	289 (65.5)	173 (63.8)	462 (64.9)	0.65
Blocked nose (*n*=726)	291 (64.7)	166 (60.1)	457 (63.0)	0.22
Joint or muscle pain (*n*=724)	231 (51.3)	110 (40.2)	341 (47.1)	0.003
Loss of taste or smell (*n*=703)	159 (36.0)	112 (42.8)	271 (38.6)	0.08
Breathing difficulties (*n*=732)	164 (36.0)	91 (32.9)	255 (34.8)	0.38
Abdominal symptoms (*n*=725)	153 (34.0)	74 (26.9)	227 (31.1)	0.046
**Number of symptoms (*n*=742)**				
⩾Seven symptoms^b^	293 (63.7)	151 (53.6)	444 (59.8)	0.006
**Duration of symptoms (*n*=741)**				
0–1 week	219 (47.7)	161 (57.1)	380 (51.3)	
2–3 weeks	188 (41.0)	99 (33.0)	281 (37.9)	
4-7 weeks	36 (7.8)	19 (6.7)	55 (7.4)	
⩾8 weeks	16 (3.5)	9 (3.2)	25 (3.4)	0.10
**Time period of symptoms (*n*=741)**				
February 2020	68 (14.8)	37 (13.2)	105 (14.2)	
March 2020	176 (38.3)	121 (43.1)	297 (40.1)	
April 2020	91 (19.8)	65 (23.1)	156 (21.1)	
May 2020	38 (8.3)	20 (7.1)	58 (7.8)	
June 2020	32 (7.0)	15 (5.3)	47 (6.3)	
July 2020	35 (7.6)	9 (3.2)	44 (5.9)	
August 2020	15 (3.3)	5 (1.8)	20 (2.7)	
September 2020	4 (0.9)	9 (3.2)	13 (1.8)	
October 2020	1 (0.2)	0 (0.0)	1 (0.1)	0.04
**Disease severity**				
Bed-bound (*n*=741)	259 (56.9)	115 (40.3)	374 (50.8)	<0.001
Hospitalised (*n*=740)	3 (0.7)	0 (0.0)	3 (0.4)	0.17

a *P* values obtained from chi^2^ test.

b The median number of symptoms was seven.

Most participants reported that their symptoms started in March or April 2020. The majority of participants had symptoms for less than 4 weeks (51.3% for 0-1 week and 37.9% for 2-3 weeks), while 10.8% had symptoms for at least 4 weeks (7.4% for 4–7 weeks and 3.4% for at least 8 weeks). Half of the participants had been bed-bound due to the disease (56.9% in women and 40.3% in men, *P*<0.001), while three participants (0.4%) had been hospitalised.

### Description of sociodemographic factors, behavioural factors, type of symptoms and self-reported testing in relation to duration of suspected COVID-19 symptoms

A comparison of participants without symptoms, short-term symptoms (<4 weeks) and long-term symptoms (⩾4 weeks) of suspected COVID-19, showed that there was no difference in the distribution of sex, occupation, daily contacts with other people during work/studies, mode of commuting to work/studies, or proportion with an overcrowded household (Table III).

**Table III.** Description of sociodemographic factors, behavioural factors and type of symptoms in relation to duration of symptoms of suspected COVID-19 (*n*=1638).

	No symptoms (*n*=897)	Symptoms <4 w (*n*=661)	Symptoms ⩾4 w (*n*=80)	*P* value^a^ all groups	*P* value^a^ <4 w vs. ⩾4 w
	*n* (%)	*n* (%)	*n* (%)		
**Female sex (*n*=1638)**	535 (59.6)	407 (61.6)	52 (65.0)	0.54	0.55
**Occupation (*n*=1635)**					
Working	463 (51.8)	343 (51.9)	38 (47.5)		
Studying	333 (37.3)	250 (37.8)	33 (41.3)		
Other	98 (11.0)	68 (10.3)	9 (11.3)	0.94	0.76
**Mode of commuting to work/studies (*n*=1638)**					
Work/studying from home	305 (34.0)	204 (30.9)	24 (30.0)		
Walking, cycling	188 (21.0)	146 (22.1)	24 (30.0)		
Car	173 (19.3)	118 (17.9)	9 (11.3)		
Public transport	224 (25.0)	188 (28.4)	23 (28.8)	0.32	0.36
**Daily contact with other people during occupation (*n*=1638)**	333 (37.1)	272 (41.2)	36 (45.0)	0.15	0.51
**Overcrowded household (>2 persons per room)** ^b^ **(*n*=1630)**	127 (14.2)	83 (12.6)	13 (16.7)	0.49	0.32
**Symptoms and testing of COVID-19**					
**Type of symptoms**					
Tiredness (*n*=736)		575 (87.7)	69 (86.3)		0.72
Runny nose (*n*=731)		482 (73.9)	50 (63.3)		0.045
Headache (*n*=730)		473 (72.7)	57 (72.2)		0.92
Sore throat (*n*=734)		469 (71.5)	54 (69.2)		0.68
Fatigue (*n*=734)		447 (68.4)	57 (71.3)		0.60
Cough (*n*=737)		431 (65.5)	57 (72.2)		0.24
Fever (*n*=712)		412 (64.8)	50 (64.9)		0.99
Blocked nose (*n*=726)		407 (62.7)	50 (64.9)		0.045
Joint or muscle pain (*n*=724)		300 (46.4)	41 (52.6)		0.31
Loss of taste or smell (*n*=703)		242 (38.7)	29 (37.7)		0.87
Breathing difficulties (*n*=732)		208 (31.9)	47 (58.8)		<0.001
Abdominal symptoms (*n*=725)		198 (30.6)	29 (37.2)		0.24
**Number of symptoms (*n*=741)**					
⩾Seven symptoms^c^		389 (58.9)	55 (68.8)		0.09
**Disease severity**					
Bed-bound (*n*=736)		331 (50.5)	43 (53.8)		0.58
Hospitalised (*n*=740)		2 (0.3)	1 (1.3)		0.21
**Tested SARS-CoV-2 IgG (*n*=1638)**	174 (19.4)	198 (30.0)	31 (38.8)	<0.001	0.11
**Positive SARS-CoV-2 IgG (*n*=399)**	6 (3.5)	56 (28.7)	9 (29.0)	<0.001	0.97
**Tested SARS-CoV-2 RNA (*n*=1638)**	39 (4.4)	119 (18.0)	20 (25.0)	<0.001	0.13
**Positive SARS-CoV-2 RNA (*n*=137)**	2 (5.1)	17 (14.5)	2 (10.0)	0.28	0.59
**Any test (SARS-CoV-2 IgG or RNA) (*n*=1638)**	193 (21.5)	272 (41.2)	45 (56.3)	<0.001	0.01
**Positive any test (SARS-CoV-2 IgG or RNA) (*n*=505)**	8 (4.2)	68 (25.4)	11 (24.4)	<0.001	0.89

a *P* values obtained from chi^2^ test.

b The definition of overcrowded household according to the National Board of Housing, Building and Planning Norm 2 (>2 persons per room excluding kitchen and living room).

c The median number of symptoms was seven.

Among participants with suspected COVID-19 symptoms, there were no major differences in type or number of symptoms when comparing the groups with long-term and short-term symptoms, except for a higher proportion with breathing difficulties among participants with long-term symptoms (Table III). No difference was observed in the proportion that were bed-bound or hospitalised during the disease. However, only three subjects had been hospitalised due to COVID-19.

Self-reported testing for COVID-19 immunoglobulin G (IgG) antibodies or active infection was reported in 31.0% of the population and was more common among participants with symptoms, especially long-term symptoms (Table III). A positive test result (IgG or active infection) was more common among participants with reported symptoms, while there was no difference in relation to length of symptoms (24.4% for long-term symptoms, 25.4% for short-term symptoms and 4.2% for no symptoms, *P*<0.001).

### Description of lifestyle and chronic diseases before the pandemic in relation to duration of suspected COVID-19 symptoms

Daily or occasional smoking before the pandemic (at the 24-year follow-up) was more common among participants with COVID-19 symptoms (26.3% for long-term symptoms, 22.6% for short-term symptoms and 15.6% for no symptoms, *P*=0.001); however, comparisons between long-term and short-term symptoms were not significant (Table IV). In contrast, there was no significant difference in the prevalence of snuff use or overweight at 24 years of age in relation to symptoms of suspected COVID-19.

**Table IV.** Description of lifestyle and chronic diseases before the pandemic (at the 24-year follow-up 2016–2019) in relation to duration of symptoms of suspected COVID-19 (*n*=1638).

	No symptoms (*n*=897)	Symptoms <4 w (*n*=661)	Symptoms ⩾4 w (*n*=80)	*P* value^a^ all groups	*P* value^a^ <4 w vs. ⩾4 w
	*n* (%)	*n* (%)	*n* (%)		
**Smoking (*n*=1636)**					
No	652 (72.8)	420 (63.4)	52 (65.0)		
Ex-smoker	104 (11.6)	91 (13.8)	7 (8.8)		
Yes, sometimes	96 (10.7)	91 (13.8)	16 (20.0)		
Yes, everyday	44 (4.9)	58 (8.8)	5 (6.3)	0.001	0.28
**Snuff use (*n*=1636)**					
No	763 (85.2)	541 (82.0)	69 (86.3)		
Ex-smoker	30 (3.4)	23 (3.5)	2 (2.5)		
Yes, sometimes	32 (5.6)	27 (4.1)	5 (6.3)		
Yes, everyday	71 (7.9)	69 (10.5)	4 (5.0)	0.39	0.35
**Overweight (*n*=1638)**	188 (21.0)	142 (21.5)	19 (23.8)	0.84	0.64
**Asthma (*n*=1633)**	104 (11.6)	75 (11.4)	14 (17.5)	0.27	0.11
**Chronic bronchitis (*n*=1618)**	40 (4.5)	38 (5.8)	8 (10.1)	0.08	0.14
**Rhinitis (*n*=1623)**	267 (30.0)	211 (32.3)	36 (45.0)	0.02	0.02
**Sensitization to inhalant allergens (*n*=1621)**	378 (42.8)	286 (43.5)	34 (42.5)	0.96	0.87
**Migraine (*n*=1600)**	126 (14.4)	139 (21.6)	28 (35.4)	<0.001	0.006
**Depression (*n*=1600)**	177 (20.2)	157 (24.4)	26 (32.9)	0.01	0.10
**ADHD or ADD (*n*=1600)**	58 (6.6)	32 (5.0)	5 (6.3)	0.40	0.61
**Consider themselves completely healthy (*n*=1627)**	601 (67.3)	398 (60.9)	35 (43.8)	<0.001	0.01
	Mean (SD)	Mean (SD)	Mean (SD)	*P* value^b^ all groups	*P* value^c^ <4 w vs. ⩾4 w
**Blood pressure (*n*=1638)**					
Diastolic (mmHg)	75.5 (8.1)	75.7 (8.2)	75.8 (7.7)	0.87	0.91
Systolic (mmHg)	121.8 (11.6)	122.5 (12.4)	121.6 (10.9)	0.42	0.49
**Lung function (*n*=1474)**					
FEV_1_ z-score	–0.25 (0.87)	–0.20 (0.85)	–0.27 (0.78)	0.54	0.54
FVC z-score	–0.06 (0.85)	–0.00 (0.86)	–0.10 (0.79)	0.37	0.35
FEV_1_/FVC z-score	–0.33 (0.89)	–0.35 (0.87)	–0.28 (0.92)	0.92	0.55

a *P* values obtained from chi^2^ test.

b *P* values obtained from analysis of variance test.

c *P* values obtained from *t*-test.

ADD: attention deficit disorder; ADHD: attention deficit hyperactivity disorder ; FEV_1_: forced expiratory volume in 1 s; FVC: forced vital capacity.

Rhinitis, migraine, depression and lower self-rated health were more common among participants with suspected COVID-19 symptoms (Table IV). In addition, rhinitis, migraine and lower self-rated health were more common among participants with long-term, compared to short-term symptoms (e.g. 45.0% vs. 32.3%, *P*=0.02 for rhinitis). Participants with long-term symptoms also had a higher prevalence of asthma, chronic bronchitis and depression; however, these differences did not reach statistical significance (Table IV). For sensitisation to inhalant allergens, ADHD or ADD, blood pressure and lung function, there were no clinically relevant differences in relation to suspected COVID-19 symptoms (Table IV).

### Comparison of self-reported tobacco use before and during the COVID-19 pandemic

The prevalence of smoking (daily or occasionally) decreased from 18.9% before the pandemic (at the 24-year follow-up) to 14.7% during the pandemic (at the current follow-up), *P*<0.001 (Table V). The decrease was largest for daily smoking, which decreased from 6.5% to 3.7%. Among daily smokers before the pandemic, 42.0% did not report current smoking, 19.6% smoked occasionally and 38.3% still reported daily smoking during the pandemic. In contrast, 6.4% of the never smokers and/or former smokers at 24 years of age reported current smoking during the pandemic (the majority occasionally) (see Supplemental Table II).

**Table V.** Comparison of self-reported tobacco use before (24-year follow-up 2016–2019) and during the COVID-19 pandemic (current follow-up 2020) in the BAMSE cohort.

	Before pandemic	During pandemic	*P* value^a^
	*n* (%)	*n* (%)	
**Smoking (*n*=1639)**			
No	1129 (68.8)	1176 (71.7)	
Former smoker	202 (12.3)	223 (13.6)	
Occasionally	204 (12.4)	181 (11.0)	
Daily	107 (6.5)	61 (3.7)	<0.001
**Snuff use (*n*=1639)**			
No	1379 (84.0)	1209 (73.5)	
Former user	55 (3.4)	67 (4.1)	
Occasionally	64 (3.9)	146 (8.9)	
Daily	144 (8.8)	222 (13.5)	<0.001

a *P* values obtained from Wilcoxon matched-pairs signed-rank test.

The prevalence of snuff use increased from 12.7% before the pandemic to 22.4% during the pandemic (*P*<0.001) (Table V). The increase was observed for both occasional and daily use. The majority (80.3%) of snuff users at 24 years continued to use snuff during the pandemic, while 14.0% of the never users and/or former users started to use snuff (7.9% occasionally and 6.1% daily). The prevalence of current snuff use was 43.9% among those who had quit smoking (daily and occasional), while the corresponding prevalence was 20.5% among current smokers.

## Discussion

In the present study based on data from a population-based cohort, we observed that almost half of Swedish young adults have had symptoms of suspected COVID-19 during the current pandemic (up to August 2020 for the majority of participants). Among these, one out of 10 have had long-term symptoms for at least 4 weeks. The observed proportion of individuals with long-term symptoms of suspected COVID-19 is in line with the COVID Symptom Study (including adults ⩾18 years, mean age 43 years), which found that 13.3% of the COVID-19 cases had symptoms for more than 28 days [5]. However, the definition of long-term symptoms of COVID-19 differs between studies [17], and the prevalence depends on the population, with a higher prevalence in severe cases such as hospitalised patients [4, 8, 18].

In the present study, we observed that symptoms of suspected COVID-19 were more common among participants who smoked before the pandemic; however, there was no significant difference in relation to the length of the symptoms. In the COVID Symptom Study, female sex, obesity and asthma were associated with long-term symptoms of COVID-19 [5], and there was a trend (although non-significant) for an association with these factors also in the present study. We further observed that long-term symptoms were more common among participants with low self-rated health and several self-reported common chronic diseases, whereas no clinically relevant differences were observed in the measured objective markers of disease (i.e. blood pressure, lung function or allergic sensitisation).

In the present study, we further observed that the prevalence of smoking had decreased, while the prevalence of snuff use had increased compared to before the pandemic (at the 24-year follow-up in 2016–2019). These changes might have several explanations. The prevalence of smoking in Sweden has decreased in the past decades [13]. According to survey results from the Public Health Agency of Sweden, the prevalence of smoking in the age group 16–29 years decreased from 17% to 13%, while snuff use increased from 15% to 18% between 2016 and 2020 [13]. It is therefore difficult to know whether the observed changes in smoking and snuff use were affected by the COVID-19 pandemic and whether these will persist over time. It is possible that the communication of smoking as a risk factor for severe COVID-19 has led to reduced smoking [19], although this might be more important for the older population. For young people, the influence of reduced social activities, parties, restaurant visits, or that people stay at home more might play a larger role, because smoking may be a more frequent social activity. Other studies on tobacco use outside of Sweden have reported mixed results in which some have observed a reduction in smoking [11, 19] and others have observed an increase in smoking during the pandemic [20, 21]. Snuff is not widely used outside of Sweden and no previous study has investigated how the pattern of snuff consumption has changed during the COVID-19 pandemic.

The main strength of the present study is the well characterised study population, with detailed longitudinal information about sociodemographic and lifestyle factors, as well as information about previous diseases and objective measures of several clinical variables such as lung function, blood pressure and allergic sensitisation. Whereas many studies have focussed on COVID-19 cases or patients [9], our study investigated the prevalence of COVID-19 symptoms in a population-based sample of young adults. A limitation is the lack of objective information on COVID-19, and only 31% of the participants reported that they had been tested for COVID-19 (antibodies and/or active infection). In the present study, we therefore investigated symptoms consistent with COVID-19, rather that the disease itself. Many of these symptoms are unspecific and are also present during other infections or diseases. For example, we observed that rhinitis, migraine and depression were more common among participant with COVID-19 symptoms, which may be partly explained by misclassification of symptoms of these diseases as COVID-19 symptoms. In addition, there may also be asymptomatic cases of COVID-19 that are not captured with questions on symptoms. However, using test results of COVID-19 only has other limitations because these results depend on the timing of the test and type of test. There may also be a selection bias if only including tested participants because these depend on the testing capacity and who has been tested. Another limitation is that the study population may not be representative of the whole country, because most participants were living in Stockholm county. During the beginning of the pandemic, the incidence of COVID-19 was higher in Stockholm compared to other parts of the country, and we may therefore observe a higher prevalence of symptoms in this study compared to other regional studies. Moreover, the study population came from households with a somewhat higher socioeconomic position compared to the original cohort. This may in contrast have introduced an underestimation of reported symptoms as in Stockholm individuals with higher socioeconomic status were less affected by COVID-19 in the beginning of the pandemic [22].

In conclusion, we observed that almost half of the Swedish young adults had experienced symptoms of suspected COVID-19 during the current pandemic. Among them, one out of 10 had symptoms for at least 4 weeks, which show that long-term symptoms of suspected COVID-19 affect a substantial proportion of young adults, although very few did suffer from a severe acute disease. Furthermore, the prevalence of smoking decreased, while the prevalence of snuff use increased during the pandemic. Our results indicate that long-term symptoms of COVID-19 may be a health concern also in young adults, and highlight the importance for policy-makers to recognise this potential health challenge to be able to plan and provide care for these patients. Future studies should focus on the potential long-term health effects of COVID-19 in young adults, particularly among those with long-term symptoms. Moreover, additional studies are needed to identify further risk factors for long-term symptoms.

## Supplemental Material

sj-docx-1-sjp-10.1177_14034948211025425 – Supplemental material for COVID-19 among young adults in Sweden: self-reported long-term symptoms and associated factors Click here for additional data file. Supplemental material, sj-docx-1-sjp-10.1177_14034948211025425 for COVID-19 among young adults in Sweden: self-reported long-term symptoms and associated factors by Sandra Ekström, Niklas Andersson, Alexandra Lövquist, André Lauber, Antonios Georgelis, Inger Kull, Erik Melén and Anna Bergström in Scandinavian Journal of Public Health
