# Supplementary material for: COVID-19 among young adults in Sweden: self-reported long-term symptoms and associated factors
Source: Scand J Public Health. 2021 Jun 19;50(1):85–93. doi: 10.1177/14034948211025425 (PMC8808012; doi:10.1177/14034948211025425)
Supplement: sj-docx-1-sjp-10.1177_14034948211025425 – Supplemental material for COVID-19 among young adults in Sweden: self-reported long-term symptoms and associated factors [file sj-docx-1-sjp-10.1177_14034948211025425.docx]

**COVID-19 among young adults in Sweden –self-reported long-term symptoms and associated factors**

Ekström S, Andersson N, Lövquist A, Lauber A, Georgelis A, Kull I, Melén E, Bergström A

**Online supplement**

**Definition of co-variables and chronic diseases**

**Baseline questionnaire (2-3 months)**

**Living area at birth:** Urban areas included central parts of Stockholm (Norrmalm). Suburban areas included northwestern parts of Stockholm county (the municipalities Järfälla, Solna and Sundbyberg).

**Parental occupation:** Parental occupation was categorized as professional worker (white collar worker) or non-professional worker (blue collar worker) according to Statistics Sweden “Socioeconomic division (SEI); Reports on Statistical Coordination 1982:4”.

**Maternal age:** Maternal age was categorized into <25 years or ≥ 25 years

**Maternal smoking during pregnancy and/or infancy**: The mother smoked at least one cigarette per day at any point of time during pregnancy and/or in infancy (yes/no).

**1-year questionnaire**

**Breastfeeding ≥ 4 months:** Exclusive breastfeeding for at least four months (yes/no).

**8-year questionnaire**

**Parental origin outside of Scandinavia:** one or both parents born outside of Sweden, Norway, Denmark, or Finland (yes/no).

**24-year questionnaire**

**Asthma** was defined from the 24-year questionnaire as doctor’s diagnosis of asthma (ever) together with symptoms of breathing difficulties or asthma medication occasionally or regularly in the last 12 months.

**Chronic bronchitis** was defined from the 24-year questionnaire as cough and mucus production in the morning during winter.

**Rhinitis** was defined from the 24-year questionnaire as symptoms from eyes or nose after exposure to furred animals or pollen (without having a flu) in the last 12 months

**Migraine, Depression and ADHD or ADD** were defined from the 24-year questionnaire as having or previously having had “Migraine”, “Depression”, “ADHD or ADD”, respectively.

**Consider themselves completely healthy** was defined from the 24-year questionnaire as answering “Completely healthy” to the question “How healthy would you say that you are?”

**24-year clinical examination**

**Overweight** was defined from measured weight and height as a body mass index ≥ 25 kg/m^2^.

**Lung function** was measured by spirometry (Jaeger MasterScreen-IOS system, Carefusion Technologies, San Diego, CA) and evaluated according to the American Thoracic Society and European Respiratory Society criteria (1) as described earlier (2). Forced expiratory volume in one second (FEV_1_), forced vital capacity (FVC) and FEV_1_/FVC ratio were analyzed using Global Lung function initiative z-scores which account for sex and height (3).

**Blood pressure:** resting systolic and diastolic blood pressure were measured three times using an automatic blood pressure meter (Omron HBP – 1300). The mean value of these measurements was used for analyses.

**Sensitization to inhalant allergens** were analyzed from the blood samples using the Phadiatop mix (cat, dog, horse, timothy, birch, mug wort, house dust mite and mold). IgE values ≥ 0.35kU/L were considered positive.

**COVID-19 questionnaire**

**Overcrowding** was defined according to the National Board of Housing, Building and Planning Norm 2 (>2 persons per room excluding kitchen and living room)

| **Table S1.** Comparison of the study population and participants in the BAMSE cohort who were not included in the COVID-19 follow-up. | | |  |
| --- | --- | --- | --- |
|  | Study population (n=1644) | Not included in the study-population (n=2445) |  |
|  | **n (%)** | **n (%)** | **P-value^1^** |
| **Male sex** (n=4,089) | 648 (39.4) | 1417 (58.0) | <0.001 |
| **Living area at birth** (n=4,071)   - Urban^2^ - Suburban^2^ | 538 (32.9)  1097 (67.1) | 667 (27.4)  1769 (72.6) | <0.001 |
| **Parental professional worker** (n=4,018) | 1378 (85.3) | 1945 (81.0) | <0.001 |
| **Maternal age <25 years** (n=4088) | 106 (6.5) | 213 (8.7) | 0.008 |
| **Maternal smoking in pregnancy and/or infancy** (n=4,086) | 188 (11.4) | 375 (15.4) | <0.001 |
| **Parental origin outside Scandinavia** (n=3,398) | 210 (14.1) | 333 (17.5) | 0.008 |
| **Breastfeeding ≥4 month** (n=3,919) | 1300 (80.9) | 1816 (78.6) | 0.07 |

1. P-value obtained from the chi-2 test
2. Urban: central parts of Stockholm (Norrmalm); suburban: northwestern parts of Stockholm county (the municipalities Järfälla, Solna and Sundbyberg).

| Table S2. Comparison of self-reported tobacco use before (24-year follow-up 2017-2019) and during the COVID-19 pandemic (current follow-up 2020) in the BAMSE-cohort | | | | |
| --- | --- | --- | --- | --- |
|  | **Current smoking (n=1639)** | | | |
|  | **No smoking** | **Occasionally** | **Daily** | **Total** |
| Smoking at 24 years | n | n | n | n |
| No smoking | 1242 | 75 | 11 | 1328 |
| Occasionally | 110 | 85 | 9 | 204 |
| Daily | 45 | 21 | 41 | 107 |
|  | **Current snuff use (n=1642)** | | | |
|  | **No snuff use** | **Occasionally** | **Daily** | **Total** |
| Snuff use at 24 years | n | n | n | n |
| No snuff use | 1233 | 113 | 88 | 1434 |
| Occasionally | 17 | 26 | 21 | 64 |
| Daily | 24 | 7 | 113 | 144 |

**References**

1. Miller MR, Hankinson J, Brusasco V, et al. Standardisation of spirometry. *Eur Respir J* 2005;26(2):319-38.

2. Wang G, Kull I, Bergström A, et al. Early-life risk factors for reversible and irreversible airflow limitation in young adults: findings from the BAMSE birth cohort. *Thorax.* Epub ahead of print 12 November 2020. DOI: 10.1136/thoraxjnl-2020-215884.

3. Quanjer PH, Stanojevic S, Cole TJ, Baur X, Hall GL, Culver BH, et al. Multi-ethnic reference values for spirometry for the 3–95-yr age range: the global lung function 2012 equations. *Eur Respir J* 2012;40(6):1324-43.
